# Editorial Chit-Chat

**Published:** 1877-07

**Authors:** 


### Editorial Chit-Chat.

QUICK WORK.—All the writing, printing, binding and mailing of THE BISTOURY has been done since the Fourth of July. The journal greets you fresh from the press, with its pages as full as ever of valuable information. Hereafter, we will be promptly on time.

PANSIES.—Would you see pansies flourish—see them grow luxuriantly, gloriously? Would you see them in all hues, colors and varieties, with markings that only nature can furnish in her lavishness? Go then, to Horseheads, peep over the garden fence into the floral department of the grounds belonging to Dr. Groom, and you will see a sight that will repay you amply for the ride. Such lovely pansies, and such quantities of them, we never saw before.

PHOTOGRAPHY.—Few artists succeed well in photographing interior views of buildings, or making good pictures of out-door scenes. We have never met but one man who can do both well, and make a picture as enjoyable and beautiful as a fine engraving. VanAken, the well known photographer of this city, is sent for far and wide to do just this sort of work, and he does it well, too. The views taken of our camping grounds, on the Lycoming, are an evidence of his wonderful skill in this sort of work. They are eagerly sought for and much prized by the visitors to that delightful retreat.

WILD FLOWERS.—Many of the wild flowers found in the woods surpass in beauty and fragrance our cultivated ones. The variety of them is surprising. One would scarcely believe that so many sorts could be obtained, until they try the experiment. They are easy of culture, too, and take kindly to a shady place, where our more pretentious flowers would not flourish at all. Among the many beautiful flowers to be found in the Institute grounds, none are more lovely or more admired than our wild-flower department. In July, you will find the dog-bane, with its beautiful and fragrant, little, lilly-shaped flowers; the meadow-rue, with its snow-flake blossoms; the evening primrose; the crane's bill; natural geranium; St. John's wurt; surrounded with ferns, star grass, and a grand array of beautiful flowering shrubs. Would you see a beautiful wild-flower garden, come to the Surgical Institute, and see what Nature can do in her own floral department.

THE CROPS.—How beautiful the fields are looking, full to the very fence-tops, with grass, wheat, corn, oats, and other cereals. Such "growing times" our farmers have not enjoyed for years. Ride out into the country, these delightful mornings and evenings, and witness the evidences of more prosperous times. Hay, wheat, corn, oats, buckwheat and potatoes will be abundant, everywhere in this region, so that our farmers will have a regular benefit.

FORGOT IT.—Just before we went to press for the last quarter, we received a natty looking little journal, styled *Aquæ Gloria.* We read it, thought it bright and spicy, and laid it carefully in our editorial drawer, for a "notice." But some rumager after what did not concern him, removed it, and so it was forgotten. Now, if our good friend, Dr. Wales, of the Water Cure, will accept this as an apology for what seemed a lack of appreciation on our part, we can say unreservedly that *Aquæ Gloria* is an institution worth the encouraging.

RAILWAY CROSSINGS.—It appears to us that, our city has grown sufficiently populous to warrant the erection of gates at several of the most important railway crossings, within the city limits. Many accidents could thus be prevented. Flagmen are well enough to warn adults, but now and then children attempt to pass before a locomotive and are caught under its wheels. Within a year, three children have been mangled in this manner, at the Hudson street crossing, while as many or more accidents have occurred on Water street. It is time that these crossings were made more secure, by erecting gates to be closed when trains are passing across our crowded thoroughfares.

HAY FEVER.—The season for this troublesome affliction is near at hand, and for the benefit of those who are in the habit of changing their locality to free themselves of it, we would advise Long Beach, opposite Tuckerton, N. J. Go to the Long Beach House, kept by Captain Bond, where you will find a delightful home, a splendid table, a dry bracing air that seems peculiarly adapted to patients of this character. Unlike any other sea air that we know of, Long Beach is a haven for hay fever sufferers. The Hotel is located upon an island, with a bay upon one side and the ocean on the other. It is well kept, delightfully situated, and has the merit of being very inexpensive. We have been there and *paid our bill*, so do not regard this as a "puff," but a matter of timely advice.

MR. TOWNER'S BOOK.—We rarely read novels,—cannot afford the time to do so; but when we *do* sit down to such a recreation, the book must be an excellent one to command our time and attention. The best declaration we can make approving of Ausburn Towner's Chedayne of Kotono is that, we have read it through from beginning to end, and at one sitting. The beauty of the book consists in the very natural behavior of its characters. They do and say things that are so perfectly man and woman-like, that you loose sight of the fiction, and, while you read, are dealing with seeming realities.

Mr. Towner has written many tales, but in Chedayne of Kotono he has placed himself upon the list of successful novelists.

---

DRAMATIC TALENT.—Our city is becoming remarkable for its dramatic and vocal talent. We can not only produce vocalists of wonderful ability, but also young people of surprising dramatic powers. The recent home entertainment given at the Opera House, in which Miss Hart, Miss Greenman and Miss DeWolfe appeared, assisted by Messrs. McCollin, Rackleyft, Gillet, Pratt, and others, delighted a large and cultivated audience of ladies and gentlemen, with their rendition of the comedy of the Big Bonanza. If these ladies and gentlemen could be persuaded to produce some of the society plays now and then, a crowded house would greet them always. We hope they will soon again appear in another play.

---

TRAMPS.—This species of the genus homo are growing so abundant and so demonstrative and expensive withal, that it is becoming a very serious question in most cities as to what shall be done to reduce their numbers or the expense of entertaining them. We think the problem is at once solved by the city authorities of Indianapolis, where all tramps and graduates of the police court, are set to work upon the streets of the city, under guard of a squad of armed police, and are required to work out the cost of their entertainment.

We should like to see the same experiment tried in our own city, believing that it would relieve us not only of the burdensome taxation consequent upon their increasing numbers, but would prove to be an inexpensive mode of working our streets.

Why not procure a steam stone crusher and convert all our highways into macadamized ones, by utilizing the innate force contained in the tramps, sixty days' criminals, and other vagabonds, supported at the public expense? Why not?

STREET GRADING.—Three weeks ago, the workmen commenced the grading of West Hudson street, in this city, by dumping a dozen or more loads of stone, at intervals of a rod, in the middle of the road. Every day or two, a fresh load is added to the unsightly mounds, until now, they have rendered the street next to impassable. Before the roadway was interfered with, we thought it quite a pleasant and smooth thoroughfare, but now, it is simply ghastly, and, what is more, seems likely to remain so for an indefinite period. If "the powers that be," or some other man, would only finish the job and relieve the denizens of the street of their misery, we, in common with the rest, will be thankful. Later: It is finished, which at once exhibits "the power of the press."

---

"JIM".—A queer chap is Jim, and the odd manœuvres to which he is addicted, challenges our respect for his intelligence, if not our approval of his pranks. Jim came to us at a tender age—when he was quite incapable of supporting himself, indeed he had not yet been weaned, and his affectionate mother parted with him with quite a demonstration of grief. But Jim did not seem to share in the apprehensiveness of his anxious parent, but came to us quite gleefully, particularly when we offered him a living trout for his meal, which we regret to say, he swallowed without the least compunction of conscience. Jim has now taken up his abode under a large, shady tree, where we can see him from our sanctum window. Even now, as we write, he is trying to unravel the mystery of the construction of Fritz's straw hat, and from the fragments strewn around, we conclude he is succeeding finely. Jim is a great lover of curiosities,—a regular connoisseur in fact, in his love for oddities. Near him lays the baby's last doll, with one of its beautiful blue eyes picked out, while a ghastly rent in its abdomen, (from which the saw-dust has all been expelled), exhibits his desire for a knowledge of anatomy. In the bark of the tree, he has fastened a particle of meat, saved from his last repast, which he is now intently watching; and, as the large black ants attempt to make their way toward it, he adroitly makes cripples of them all, while he watches their hobbling with the satisfaction of a manufacturer of apparatus for the deformed. Jim has opened a museum, which he guards with jealous care, and if the thimble, scissors, or other useful article of the matron is lost, there it is sure to be found. Jim can talk—Jim can sing,—not melodiously, to be sure, but then he sings,—Jim is a buster,—Jim's a crow.

PLATO'S BEST THOUGHTS.—This is a book of nearly 500 octavo pages, compiled from Prof. Jewett's translation of the dialogues of Plato, by Prof Bulkley of Boston. The book is a valuable one to professional, and other men of learning, who value the precious thoughts of the famous and learned Greek Philosopher. In this compilation, rare good judgment is exercised in the selection of themes treated upon, so that the very finest passages, which classical scholars are so fond of quoting from, in Plato's Dialogues, are herein given. It is rare good reading for any one of cultivated taste. Scribner, Armstrong & Co., Publishers, New York.

---

CITY LABORERS.—In the street, before our house, has the city employed several workmen, to do the grading. They are a queer lot of chaps that are there employed. We have been watching their movements for the past hour, and must accord to them a skill in deliberate movement, not attained by any other class of workmen within our knowledge. Two men have been trying to occupy the forenoon in cleaning the grass from a gutter that is just fifty feet long. Would you believe it,—they employed themselves constantly and at their nooning, the job remained but half completed! The chap with the shovel, deposited exactly fifteen shovels full of earth, from the gutter to the street, in forty-five minutes, by the watch. The fellow with the pick, was not quite so skillful, as he made six and a half motions with his pick, and then had to resort to the subterfuge of lighting his pipe three times, and went to the pump for a drink of water twice, before he got away with the same number of minutes. For this, they are paid one dollar and a quarter a day, and their labor denominated "unskilled." We protest against the term. No class of workmen could begin to consume as much time as that, and be constantly employed,—not accomplishing anything, without possessing the highest order of merit and skill.

Should the present cool, delightful weather continue, we estimate that, with average good luck, these two skilled city laborers will get across our one hundred and thirty foot lot, in about three days, with a saving to the city that can scarcely be estimated.

---

CATARACT OPERATIONS.—A recent report upon the success of the operations for cataract in the Vienna Cliniques, by Profs. Arlt, Becker, and others, than whom no better operators exist in all Europe, we find that in 100 cases, 15 resulted badly. In 217 other cases, 117 progressed favorably, and 45 were accompanied by more or less serious accidents.

This is regarded as successful operating. Certainly, no better operators exist anywhere than the two very distinguished Professors mentioned, and it may be in bad taste for us to compare results with these gentlemen, but the difference is so great that we confess to a little pride in the matter. Out of 428 operations for cataract performed in the Elmira Surgical Institute, but 7 failures have occurred. In the last 68 cases, but one failure came to us, and that from non-union of the wound and consequent sloughing of the cornea, in an old gentleman not in the best of health.

It may do for Professors in large and celebrated hospitals to fail in half their operations, but it would never answer for a private practitioner,—his reputation would be utterly ruined.

Perhaps the success attending our operations depends more upon the healthful location,—a well ventilated and not over-crowded hospital, with pleasant surroundings, and to the after-treatment, under the observation of the operators, as to any other circumstance. At all events, for any surgical operation, we would prefer a country hospital to one in any large city.

---

OUR DELAY.—As was intimated in our last number, we find ourselves late this quarter, by reason of our stay in the woods. Our friend H., and others, assisted us in our primitive mode of life, and did much to make our vacation a delightful one. We pitched our tents upon the same old spot, upon the banks of the beautiful Lycoming Creek, where we rested in the cool shade, at midday, and cast the fly in the early morning, for the sprightly trout.

Nothing can be more refreshing to a weary body and mind than a month's stay in the woods, where all is peace and quietness. How glorious the mornings are,—how sweetly the birds sing, waking you long before the sun finds his way over the hill-tops, with their sweet melody! Then, when George sounds the horn for breakfast, how eagerly we respond to the summons, first having bathed our face in the clear, rippling water that invites us to the ablution. With what satisfaction we fill our lungs with the sweet, morning air, and, striking our closed hands upon our chest, smile at the resonance of the blow, and the evidence of a sound, wholesome respiration! Then, with what a gusto we partake of the morning meal,—did pancakes, honey, coffee, boiled potatoes and roasted trout *ever* taste so well before? No,

*never*, neither could such a breakfast be partaken of and so thoroughly enjoyed without the glorious surroundings.

Breakfast through with, how eager were we all to test our skill upon the wary trout that were leaping from the several beautiful pools within sight of breakfast table, and many were the wagers made as to who should deliver the finest trophy of the rod, for the midday meal. How H. and the Doctor vied with each other in the jolly contest, first bringing in their ten inch trout, then twelve and thirteen inches, until a "monster," measuring fifteen inches and weighing a pound and five ounces, arrested further contest in that direction. Did you ever capture a fifteen-inch trout, with a delicate fly, upon a slender leader, and an elastic rod, weighing less than seven ounces? Did you? Fun! Shade of Izaak Walton, fun is no name for i. Imagine a beautiful, clear morning, with the sun just peeping over the high mountains with which the valley is surrounded;—a clear pool, upon which no ripple occurs, save those that roll in gentle rings from bank to bank as each succeeding step places you nearer to the overhanging bush under which your trout is supposed to lay. Carefully you make your cast, landing your flies but a few feet from the spot; and, as you seek to recover your line to make another cast, a whirl in the water assures you that your expected fish is there, but has missed the fly presented to him. From his wake, you determine him to be a rouser, and stand almost motionless, while you reel in your line for another cast. Throwing your flies through the air, back and forth until the required length of line has been again unreeled, the gossamer leader with its downy flies, shoot directly toward the spot, and as the curve in the line uncoils, the leader is shot in a straight line immediately over the spot, where the deceptive flies settle upon the water softly, gently, without the slightest splash to indicate their position. Suddenly the water boils, sending a thrill through your body and an involuntary movement to the wrist, and as the line becomes taut, the dull thud, as you strike, gives evidence that your game is hooked, and that he is the largest of his kind. Instantly your reel is set to whizzing, as the fish rushes madly up the pool, while every inch of line is taken in his flight, as the terrific strain upon the rod bends it pliantly, from tip to butt; and, as he feels the stout resistance to his advance, he leaps from the water and throws the crystal drops from his golden sides and wildly shakes his head to free himself from the hook. You are careful that he secures no slack, and cautiously reel him towards you. Then he sulks at the bottom of the pool, remaining so immovable that you think he has fastened your hook to a root and made good his escape. The thought sends a cold chill down your spine, while you carefully draw upon the line to ascertain whether he has really fled. Soon, your pressure has the desired effect of bringing him again to the surface, where he flounders and dives to and fro in a manner that tests your tackle to the utmost. As you reel him near to you, the landing net is gotten ready for his reception, but before you can get it under him, away he goes again, down the pool, until he rests upon the very verge of the rapids. Your chances now of saving the prize are precarious in the extreme, and while the trout struggles and tugs at your line to gain the swift water and so elude your hold upon him, you turn the butt of the rod toward him that he may have the full spring of it, and then commences a contest of skill decidedly in favor of the fish. Oh, how he *does* pull, and when he shoots across the the riff, the water hisses as it is cut by the fine line that throws it into beautiful, sparkling spray, in the sunlight. Again and again he breaks, leaping far into the air, while you shout like a Modoc at every jump. Presently, you secure a stone from the bottom of the creek and hurl it with all your might below the spot where the trout is struggling, —it has the desired effect, and up the pool he rushes again, compelling you to quicken your pace in the same direction, while you reel in your line that no slack may count against you. Now he attempts to reach a tree-top that is trailing in the water, and sets your nerves to tingling as he shoots by it, missing it by an inch. Then he essays to circumnavigate an old stump, farther on, all of which laudable desires on his part you skillfully frustrate. Presently, he becomes tired, his plunges grow less frequent, and his side-long skips less alarming, until you are able to reel him close to you, where he lies upon the surface of the water, and gives up the contest, while you carefully slip the net under him and carry him triumphantly to shore. Then it is that you make the hills ring with your triumphant shout, that brings a like response from your friends in camp, who run to meet you as you bear your trophy in. How you enjoy the triumph as the campers comment upon the beauty of the fish, and with what joy you relate the incidents of his capture, about the campfire at night.

This is but one of the many enjoyments we experienced in our recent vacation, from which we have just returned, invigorated in health and as brown as a berry.

